# Nosocomial infection among patients with COVID-19: A retrospective data analysis of 918 cases from a single center in Wuhan, China

**DOI:** 10.1017/ice.2020.126

**Published:** 2020-04-13

**Authors:** Yan He, Wei Li, Zhen Wang, Huilong Chen, Lei Tian, Dong Liu

**Affiliations:** 1 Department of Pharmacy, Tongji Hospital Affiliated to Tongji Medical College, Huazhong University of Science and Technology, Wuhan, China; 2 Department of Infectious Diseases, Tongji Hospital, Tongji Medical College, Huazhong University of Science and Technology, Wuhan, China; 3 Department of Microbiological Laboratory, Tongji Hospital, Tongji Medical College, Huazhong University of Science and Technology, Wuhan, China

*To the Editor—*The emergence of coronavirus disease-2019 (COVID-19) in China at the end of 2019 has caused a global pandemic and is a major public health issue.^1^ The percentage of nosocomial infection among COVID-19 patients who have died was significantly higher than that of patients who were cured and discharged (*P* = .002).^2^ We investigated nosocomial infection among COVID-19 patients, and we analyzed risk factors to provide basic data for nosocomial infection prevention and control.

We retrospectively analyzed the clinical data of 918 COVID-19 patients in Tongji Hospital from December 30, 2019, to February 29, 2020. We performed a 1:4 paired case-control study: 65 patients with nosocomial infection were assigned to the case group and 260 non–nosocomial infection patients were assigned to the control group. We analyzed clinical data regarding patient demographics, basic disease, and treatments, and we summarized influencing factors of NI among COVID-19 patients. This study was approved by the Ethics Commission of Tongji Hospital (no. TJ-IRB20200338).

Male gender accounted for 47.7% of the 65 patients in case group (Table 1). The median age at the time of admission was 51 years (IQR, 36–71 years). In total, 40 of 65 patients (65.6%) in the case group had comorbidities; the most prevalent of these were hypertension (36.9%), cardiovascular disease (18.5%), and diabetes (18.5%). Approximately one-third of patients (33.8%) had COVID-19 at the time of admission.^3^ The nosocomial infection rate among COVID-19 patients was 7.1% (65 of 918). The most common nosocomial infection was pneumonia (32.3%), followed by bacteremia (24.6%), and urinary tract infection (21.5%). In total, among the 43 pathogens isolated from nosocomial infections, 17 were gram-positive bacteria, 21 were gram-negative bacteria, and 5 were fungi. Onset of nosocomial infection occurred as early as day 7 of the course of illness and as late as day 22, with an average of 14.3 ± 8 d. The mortality of COVID-19 patients with nosocomial infection was 15.4%, significantly higher than that of COVID-19 patients without nosocomial infection (7.3%; odds ratio [OR], 3.87; 95% confidence interval [CI], 0.84–4.16; *P* = .045).

**Table 1.** Characteristics of Nosocomial Infection Among Patients With COVID-19

Characteristic	No. (N=65)	%
Age, median y (IQR)	51 (27–68)	
**Sex**		
Male	31	47.7
Female	34	52.3
**Comorbidities**	40	65.6
Hypertension	24	36.9
Cardiovascular disease	12	18.5
Diabetes	12	18.5
Underlying hematological disease	3	4.6
Chronic kidney disease	3	4.6
Respiratory disease	2	3.1
Cerebrovascular disease	2	3.1
Chronic liver disease	1	1.5
Malignancy	1	1.5
**Charlson comorbidity score**		
2	46	70.8
3–4	19	29.2
**Clinical classification of COVID-19**		
Severe type	43	66.0
Critical type	22	33.8
**Invasive devices (CVC or PICC)**	25	38.5
**Prophylactic application of antibiotics**	49	75.4
Cephalosporins	6	9.2
Fluoroquinolones	40	61.5
β lactam/β-lactamase inhibitors	5	7.7
Azithromycin	3	4.6
Ornidazole	2	3.1
Combination of antibiotics^a^	7	10.8
**Infection site**		
Pneumonia	21	32.3
Bacteremia	16	24.6
Urinary tract infection	14	21.5
Skin soft-tissue infection	8	12.4
Gum infection	4	6.2
Others	2	3.1
**Antiviral treatment**	46	70.8
**Glucocorticoid treatment**	25	38.5
**Pathogen isolates**	43	
*Coagulase negative staphylococcus*	12	27.9
*Acinetobacter*	9	20.9
*Pseudomonas aeruginosa*	6	14.0
*Enterococcus faecium*	5	11.6
*Klebsiella pneumoniae*	4	9.3
*Escherichia coli*	2	4.6
*Candida albicans*	2	4.6
*Mucor*	2	4.6
Other	1	2.3
**Mortality**	10	15.4

Note. IQR, interquartile range.

The association between demographic and clinical factors and the treatment of nosocomial infection as determined by univariate and multivariable analyses was displayed in Supplementary Table 1. Significant positive associations between nosocomial infection and the following were detected by univariate analysis: diabetes, hematological disease, invasive devices (central venous catheter [CVC] or peripherally inserted central catheter [PICC]), combination of antibiotics, and glucocorticoid treatment. Among these factors, the highest odds ratio was for invasive devices (OR, 4.62; 95% CI, 2.47–8.62) followed by diabetes (OR, 3.04; 95% CI, 1.38–6.69), combination of antibiotics (OR, 3.02; 95% CI, 1.10–8.26), glucocorticoid treatment (OR, 2.44; 95% CI, 1.36–4.37), and hematological disease (OR, 1.95; 95% CI, 1.01–1.06).

For multivariable analysis, the dependent variable was nosocomial infection status and independent variables were all factors that demonstrated statistical significance, as mentioned with univariate analysis. Significant predictors of nosocomial infection after adjustment for other covariates were invasive devices (OR, 4.28; 95% CI, 2.47–8.61; *P* =.007) followed by diabetes (OR,: 3.06, 95% CI, 1.41–7.22; *P* =.037), and combination of antibiotics (OR, 1.84, 95% CI, 1.31–4.59; *P* = .003) (Supplementary Table 1 online).

In conclusion, these findings suggest that nosocomial infections are common among patients with COVID-19 and can be predicted by considering certain risk factors. Rational utilization of antibiotics and steroids to treat patients with COVID-19 is important in preventing nosocomial infection, and special attention should be given to diabetic patients and patients with invasive devices (ie, CVC or PICC). Future studies are warranted to evaluate the efficacy of implementing infection control strategies or protocols on COVID-19 patients to achieve better therapeutic outcomes.
